# Anuria, an Atypical Presentation of Leptospirosis: A Case Report

**DOI:** 10.31729/jnma.6240

**Published:** 2022-01-31

**Authors:** Shambhu Khanal, Biraj Pokhrel, Madalasha Pokhrel, Rukshar Thapa, Rabin Nepali

**Affiliations:** 1 Department of Internal Medicine, Tribhuvan University Teaching Hospital, Maharajgunj, Kathmandu, Nepal; 2 Maharajgunj Medical Campus, Institute of Medicine, Tribhuvan University, Maharajgunj, Kathmandu, Nepal; 3 Department of Internal Medicine, Manipal College of Medical Sciences, Pokhara, Nepal; 4 Department of Nephrology, Tribhuvan University Teaching Hospital, Maharajgunj, Kathmandu, Nepal

**Keywords:** *anuria*, *case report*, *leptospirosis*, *renal insufficiency*, *zoonoses*

## Abstract

Leptospirosis, an underreported disease, is a highly prevalent spirochaetal zoonotic disease in both tropical and temperate climates. Symptoms can range from mild illness to potentially life-threatening infection. Laboratory tests are nonspecific. Microbiological confirmation is not widely available in endemic developing countries like Nepal. We need to rely on the serologic test, which has its own pitfalls in the initial days of illness. Here, we report a case of 56 years old female from the western region of Nepal who presented with fever, jaundice and anuria. She initially tested negative for leptospirosis but was later found to be positive in the second week of illness. Unlike the usual non-oliguric renal failure in leptospirosis, she presented with anuria requiring haemodialysis and subsequently had a good recovery with treatment. We highlight the importance of clinical suspicion and logical interpretation of serologic tests based on its timing from the onset of illness.

## INTRODUCTION

Leptospirosis is a spirochaetal zoonotic disease prevalent in both tropical and temperate climates. The most notable source of infection for humans is brown rat, highly contagious, especially in rainy seasons in areas of poor sanitation after direct or indirect exposure to infected animals shedding Leptospira in their urine.^1^ Patients present with the self-limiting anicteric, nonspecific flu-like illness presenting as fever, chills, headache, lethargy, and myalgia in 90% of the cases whereas jaundice, acute renal failure, and respiratory distress in about 10% of the cases.^2^ Leptospirosis needs to be strongly suspected if farmers presents with these symptoms from endemic area.

## CASE REPORT

A 56-year-old female from western Terai presented to our emergency department in September with a history of fever for five days, the maximum temperature documented 102° Fahrenheit, associated with chills, rigor, backache and polyarthralgia. She also complained of progressive yellowish discoloration of eyes, darkening of the colour of urine and mild to moderate abdominal pain on the right upper quadrant region. It was not associated with itching or clay coloured stool. Her urine output decreased amounting to oliguria, three days after the onset of fever which then progressed to anuria in the following days.

She did not have a history of use of Non-steroidal antiinflammatory drugs (NSAIDs) or alternative medicines, prior history of nausea, vomiting, loose motions, neck stiffness or altered mental status.

On her initial presentation, she was ill-looking and had pallor as well as icterus. There were no signs of clubbing or lymphadenopathy. She had a firm, tender liver measuring three centimetres below the right subcostal margin in the midclavicular line. There was no splenomegaly. The rest of the systemic examinations were unremarkable.

Her laboratory parameters at the time of admission are given below. Her liver function test showed conjugated hyperbilirubinemia (Table 1).

**Table 1:** Laboratory parameters of the patient at the time of admission and discharge with the reference value.

Parameters	Obtained value (at the time of admission)	Obtained value (at the time of discharge)	Reference value
Total leucocyte count	30 × 10^3^/mm^3^	12.7 × 10^3^/mm^3^	4,000-11,000/mm3
Neutrophils	70%	Not available	<60%
Lymphocytes	20%	Not available	<20%
Hemoglobin	8.3g/dl	8.54g/dl	14-18g/dl
Platelet count	78 × 10^3^/mm^3^	420 × 10^3^/mm^3^	150,000-450,000/mm^3^
Creatinine	692 μmol/L	355 μmol/L	62-115 μmol/L
Blood Urea Nitrogen (BUN)	20mmol/L	15mmol/L	8 - 20mmol/L
Sodium	123meq/L	139meq/L	136 -145meq/L
Potassium	3.6meq/L	3.7meq/L	3.5 - 5meq/L
Total bilirubin	620 μmol/L	46 μmol/L	5.1 - 17.1 μmol/L
Direct Bilirubin	210 μmol/L	21 μmol/L	1.7 - 5.1 μmol/L
Aspartate transaminase	149U/L	19U/L	10 - 40U/L
Alanine transaminase	94U/L	6U/L	10 - 40U/L
Alkaline Phosphatase	505U/L	258U/L	30 - 120U/L
Albumin	25g/L	Not available	35 - 55g/L

She tested negative for Leptospira IgM antibody test initially. However, we tested her again for the Leptospira IgM antibody owing to a strong clinical suspicion. She tested positive on the repeat test that was done ten days after the onset of illness. Based on the initial negative report of leptospirosis, she was treated primarily with a suspicion of sepsis but the etiological diagnosis was obscure. She tested negative for Malaria, leishmaniasis, scrub typhus, and brucella.

She was treated in the intensive care unit and her lab reports were followed frequently. She showed no clinical improvement with ceftriaxone, which she received for 5 days before visiting us. We empirically treated her with meropenem one gram intravenously three times a day and doxycycline 100mg intravenously two times a day for ten days. Due to the persistent anuria and progressive rise in serum creatinine, she was started on haemodialysis. She showed drastic improvement in her liver function test. Her laboratory parameters at the time of discharge are mentioned above in (Table 1).

Despite the clinical improvement on jaundice, she did not show much improvement in urine output. She remained anuric for about a month after which her urine output gradually increased. She was then discharged after a month of hospital stay with the advice of continuing haemodialysis twice a week. After three months of the onset of illness, she no longer required dialysis and her renal function showed serum creatinine of 226jmol/L. On the fourth month of follow up, her renal function was completely restored to normal with a serum creatinine of 88 μmol/L.

## DISCUSSION

Leptospirosis is a neglected zoonotic disease, caused by aerobic spirochete namely Leptospira sp. and is endemic in tropical countries. Rodents are the natural reservoirs and source of transmission while humans are the accidental hosts. The portals of entry are cut or abrasion on the skin and mucous membrane of the conjunctiva and oral cavity. Leptospira disseminates via the bloodstream in the body without forming any skin lesions unlike other spirochetes and settles in renal tubules and peritubular capillaries because of a special affinity for them.^1^ Evidence shows that the cytokine storm with the release of IL-10, TNF-alpha occurs in severe leptospirosis when there is a high level of leptospiraemia. The high bacterial load and dysregulated host response explain the multiorgan failure and sepsis in leptospirosis.^3^ The incubation phase averages from seven to twelve days, though it can be as short as three days or as long as a month.^1^ The patient usually present with wide range of symptoms ranging from mild fever, headache, back pain, lethargy, and myalgia, to severe symptoms presenting with multiorgan failure with a major target of kidney, liver, and lungs, popularly known as Weil's syndrome. Conjunctival suffusion is a pathognomonic feature of leptospirosis.^1,2^

Leptospirosis has a special predilection for proximal renal tubules, which has been attributed to the peculiar characteristic of non-oliguric renal failure with or without hypokalemia.^4^ Acute renal failure in leptospirosis usually takes five to eight days to recover. Significant improvement in urine output after adequate hydration is observed in oliguric renal failure. Seguro et al showed that 46% of oliguric patients at admission will become non-oliguric after volume replacement and use of furosemide.^5^ Similarly, patients with non-oliguric renal failure had a better prognosis than oliguric renal failure in leptospirosis. The common contributing factors for the renal failure are dehydration, rhabdomyolysis and high bilirubin. However, the renal function is restored. Dialysis dependence is very rare and only nine percent of cases show features of early-stage CKD.^6^ It takes usually six months for full recovery of GFR, whereas, urine concentrating ability may take a longer time.^7^ In contrast to usual findings, our patient had anuria at the time of presentation, required haemodialysis for life support and eventually had a full recovery of GFR in three months.

IgM ELISA is used for screening and confirmation of acute leptospiral infection but requires an adequate number of detectable antibodies which are produced after five to seven days. The molecular tests such as PCR are better at detecting leptospirosis in acute septicaemic phase, which occurs in three to seven days of infection. However, they are not widely available tests. So, it is mainly a clinical diagnosis in the acute septicaemic phase and the timing of antibody-based ELISA test should be cautiously noted. The rapid diagnostic IgM ELISA may provide false negative results in the first week of illness as it was in our patient. Such an imprudent interpretation of serologic test delays the diagnosis, specific treatment and recovery.^8^ Provided that clinical background and serologic tests are wisely taken into account together, rapid ELISA tests and immunochromatography are rapid, cost-effective and highly sensitive screening tests in low-resource settings.^9^ In severe leptospirosis, combined treatment with doxycycline and cephalosporin, cefotaxime would be the better choice than doxycycline alone. Besides, doxycycline is a cheap and effective antibiotic for an empirical treatment in endemic areas where leptospirosis is a common clinical suspicion.^10,11^ Leptospirosis is an underappreciated and neglected tropical disease. It should be a common and foremost differential diagnosis in an endemic area when a patient presents with fever, jaundice, and anuria even if the antibody based serologic test is negative.
